# An In Vivo Study of Low-Dose Intra-Articular Tranexamic Acid Application with Prolonged Clamping Drain Method in Total Knee Replacement: Clinical Efficacy and Safety

**DOI:** 10.1155/2015/164206

**Published:** 2015-10-25

**Authors:** Paphon Sa-ngasoongsong, Pongsthorn Chanplakorn, Siwadol Wongsak, Krisorn Uthadorn, Tanapong Panpikoon, Paisan Jittorntam, Katcharin Aryurachai, Pantap Angchaisukisiri, Viroj Kawinwonggowit

**Affiliations:** ^1^Department of Orthopedics, Faculty of Medicine Ramathibodi Hospital, Mahidol University, Bangkok, Thailand; ^2^Department of Radiology, Faculty of Medicine Ramathibodi Hospital, Mahidol University, Bangkok, Thailand; ^3^Section of Research, Education, and Innovation, Faculty of Medicine Ramathibodi Hospital, Mahidol University, Bangkok, Thailand; ^4^Department of Medicine, Faculty of Medicine Ramathibodi Hospital, Mahidol University, Bangkok, Thailand

## Abstract

*Background*. Recently, combined intra-articular tranexamic acid (IA-TXA) injection with clamping drain method showed efficacy for blood loss and transfusion reduction in total knee replacement (TKR). However, until now, none of previous studies revealed the effect of this technique on pharmacokinetics, coagulation, and fibrinolysis. *Materials and Methods*. An experimental study was conducted, during 2011-2012, in 30 patients undergoing unilateral TKR. Patients received IA-TXA application and then were allocated into six groups regarding clamping drain duration (2-, 4-, 6-, 8-, 10-, and 12-hours). Blood and drainage fluid were collected to measure tranexamic acid (TXA) level and related coagulation and fibrinolytic markers. Postoperative complication was followed for one year. *Results*. There was no significant difference of serum TXA level at 2 hour and 24 hour among groups (*p* < 0.05). Serum TXA level at time of clamp release was significantly different among groups with the highest level at 2 hour (*p* < 0.0001). There was no significant difference of TXA level in drainage fluid, postoperative blood loss, blood transfusion, and postoperative complications (*p* < 0.05).  *Conclusions*. Low-dose IA-TXA application in TKR with prolonged clamping drain method is a safe and effective blood conservative technique with only minimal systemic absorption and without significant increase in systemic absorption over time.

## 1. Introduction

Total knee replacement (TKR) is a major orthopaedic operation that significantly associates with large amount of perioperative blood loss (PBL) and the need of blood transfusion. Therefore, perioperative blood loss management, in order to prevent bleeding related complication and transfusion-related morbidity [1, 2], is one of the most important factors for successful postoperative outcome. Regarding the proven methods used for reducing PBL in TKR, intra-articular tranexamic acid (IA-TXA) application together with 1-hour or 2-hour drain clamp has been demonstrated as ability to decrease PBL and proportion of patients requiring postoperative blood transfusion [3–8]. However, there was no consensus regarding the time that drain should be clamped for maximum benefit of intra-articular clot stabilization and minimize systemic absorption, and the recent studies also supported that prolonged clamping drain up to 12 hours is safe and effective for blood loss reduction [9–13]. Through our knowledge, none of the previous studies has been shown about pharmacokinetics of tranexamic acid (TXA) within joint space after intra-articular application with drain clamping in TKR and its effect on systemic homeostasis. Therefore, this study aimed to evaluate the serum TXA level and the TXA concentration in drainage fluid after IA-TXA with specific time after drain clamp, in order to understand the pharmacodynamics of TXA within joint space and ability of systemic absorption and correlate with the other related postoperative outcomes.

## 2. Patients and Methods

A prospective experimental study was conducted, between 2011 and 2012, in 30 patients who underwent unilateral TKR in Ramathibodi Hospital. The study was approved by Committee on Human Right Related to Researches Involving Human Subjects (Protocol number ID 09-54-28). Informed consent was obtained from all participants, before the surgery was scheduled in accordance with the Declaration of Helsinki.

The inclusion criteria were (1) the patients who diagnosed as primary knee osteoarthritis and underwent primary unilateral cemented conventional TKR and (2) osteoarthritis grades II-III according to Ahlbäck classification [14]. The exclusion criteria were (1) risk of abnormal bleeding tendency or bleeding disorder (normal coagulogram, serum creatinine < 2.0 mg/dL, stopping nonsteroidal anti-inflammatory drugs and antiplatelet drugs more than 7 days), and (2) contraindication for TXA use (active intravascular clotting process, acquired defective colour vision, subarachnoid hemorrhage, hypersensitivity to TXA, and any of history of serious adverse effects, thrombotic disorder, and hematuria).

The blocked-randomization was generated by STATA 11.0 software (Stata Corp, College Station, Texas, USA) and further concealed with sealed envelopes in the sequentially numbered container. The surgery was performed by one of the authors (Viroj Kawinwonggowit), who was an experienced arthroplasty surgeon, under spinal anesthesia. The prostheses used in this present study were Nexgen total knee system (Zimmer Inc., Warsaw, Indiana, USA). Due to awareness of sex-related difference in anteroposterior diameter of the distal femur, Nexgen Gender prosthesis was specifically used in female patient while Nexgen Flex prosthesis was used in male patient. The pneumatic tourniquet was applied at proximal thigh with pressure as 350 mmHg. The surgical approach was midline skin incision, medial parapatellar arthrotomy, and midvastus incision. After the bony structure was prepared, all prosthesis components were inserted with full cementation (Palacos, Hevaeus Medical GmbH, Germany). Two standard drain tubes (size 8 Redon drain, B-Braun Ltd.) were placed deep into knee joint, which exited superolaterally. No superficial drain was used in this study. One drain tube was used to apply 500 mg intra-articular tranexamic acid injection and then connected to vacuum drains (Drainobag 600V Lock, B-Braun, Melsungen AG, Germany) [7]. Another drain was connected to smaller vacuum drains (Drainobag 150V Lock, B-Braun, Melsungen AG, Germany) in order to collect the drainage fluid to measure TXA level. Subcutaneous and skin closure was performed subsequently. Bulky compressive dressing was applied and both drains were clamped before tourniquet was deflated.

After the operation, the research assistant, who was not involved with the surgery, was responsible for opening the envelopes, opening the drain at the setting time, and recording the amount of drain volume as data collection protocol. Then the patients were randomly allocated into six groups regarding clamping drain duration (2-, 4-, 6-, 8-, 10-, and 12-hour). Postoperative blood samples were collected preoperatively, at the time of clamp release, 24-hour, 3-day, and 2-week postoperatively. Drainage fluid was collected at the time of clamp release by one of the authors (Krisorn Uthadorn) by standard protocol. The clamp of the smaller vacuum drain was released first to collect 50 mL of drainage fluid and then this drain was removed. Then, the clamp of larger vacuum drain was then fully opened later. Standard postoperative care protocol was applied to all patients. Blood transfusion was considered according to American Society of Anesthesiologists (ASA) guideline [15]. The amount of blood loss and transfusion was recorded. All patients were sent to document deep vein thrombosis (DVT) by duplex ultrasound, performed by an experienced radiologist (Tanapong Panpikoon) on the fourth postoperative day. If the patients who developed postoperative clinical presentation suspected pulmonary embolism (PE) such as acute dyspnea or unexplained hypoxemia, the computer tomographic angiogram was then performed to confirm the diagnosis by the same radiologist. All patients were followed for clinical outcome for one year.

Patients' demographic data and preoperative laboratory values were collected. Serum TXA level at 2 hours, time of clamp release, and 24 hours postoperatively and drainage TXA level were measured with mass spectrometry [16, 17], by one of the authors (Paisan Jittorntam). Total hemoglobin loss (THL) was calculated from the difference between preoperative Hb and postoperative Hb on the third postoperative day. Calculated total blood loss (CTBL) was calculated by using specific method [18, 19].

Blood samples were taken from all patients for measuring the coagulation and fibrinolytic markers with the standard technique using calibrated machine (as platelet count, prothrombin time (PT), partial thromboplastin time (PTT), thrombin time (TT), international normalized ratio (INR), D-dimer, and fibrinogen) at the time before operation, 2 hours, time of clamp release, 24 hours, 3 days, and 14 days postoperatively. Specific fibrinolytic markers, as plasmin inhibitor (PI), plasminogen (PLG), plasminogen activator inhibitor type 1 (PAI-1), and tissue plasminogen activator (t-PA) were all measured with standard kit, by one of the authors (Katcharin Aryurachai), at, preoperatively, 2 hours, time of clamp release, 24 hours, and 3 days postoperatively (tPA (Asserachrom tPA kit; Diagnostica Stago, Asnières-Sur-Seine, France), Plasma PAI-1 (Asserachrom PAI-1, Diagnostica Stago), Plasmin inhibitor (HemosIL Plasmin inhibitor, Instrumentation Laboratory), and Plasminogen (HemosIL Plasminogen, Instrumentation Laboratory)).

Statistical analysis was performed using Stata software version 11.0 (Stata Corp, College Station, Texas, USA). Normality of data was tested by Kolmogorov-Smirnov test. Continuous data were presented as mean and standard deviation and compared with one-way analysis of variance (ANOVA). The comparison of serial laboratory values between groups according to time was performed by repeated measurement with Scheffe test post hoc analysis. Categorical data were presented as proportion and compared with Chi-square test.

## 3. Results

A total of 30 patients (8 males and 22 females) were recruited into this study and then were allocated into six groups according the blocked randomization. The average age and BMI were 69.7 ± 6.9 years (range 50–78 years) and 26.8 ± 2.9 kg/m^2^ (range 22.5–33.3 kg/m^2^). The mean preoperative Hb was 13.0 ± 1.4 g/dL (range 10.7–15.6 g/dL). There was no significant difference in gender, age, height, weight, BMI, ASA physical status, side of operation, and preoperative laboratory values among groups as shown in Table 1.

**Figure 1:**
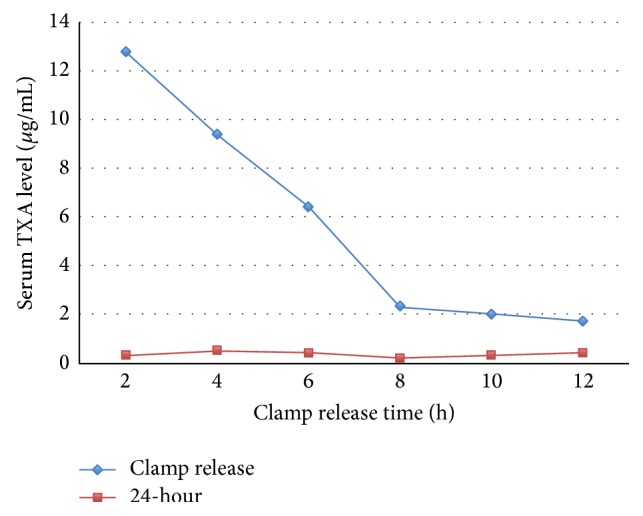
Serum tranexamic acid (TXA) level at time of clamp release and 24 hours in each group.

There was no significant difference between groups in serum TXA level at 2 hours and 24 hours postoperatively; however, the serum TXA level at the clamp release time was significantly lower according to time period of drain clamp (Figure 1) (*p* < 0.0001). The TXA concentration in drainage fluid was not significantly different between groups as shown in Table 2. No significant difference was found in DBL, THL, and CTBL between groups (Table 3).

No significant difference was found between groups in platelet count, PT, PTT, TT, INR, D-dimer, fibrinogen, PI, PLG, and t-PA preoperatively (*p* > 0.05); however there was a significant difference between groups in preoperative PAI-1 (*p* = 0.01). After surgery, there were significant differences between groups in D-dimer (14 d, *p* = 0.01), fibrinogen (time of clamp release, *p* = 0.02), PAI-1 (time of clamp release, 24 hours, and 3 days, *p* = 0.002, 0.03, and 0.03 resp.), and t-PA (2 hours and time of clamp release, *p* = 0.05 and 0.02) (Table 4).

Neither deep vein thrombosis nor infection nor wound complication was found immediately after operation and during 1-year follow-up period.

## 4. Discussion

Regarding the perioperative blood loss management in TKR, intra-articular tranexamic acid (IA-TXA) application has recently become a popular method due to its excellent efficacy for reducing PBL and transfusion requirement. However, through our knowledge, previous studies demonstrated ability to reduce postoperative blood loss with a variety of techniques and mostly using 1-2-hour clamp drain technique [3–8]. Moreover, several recent studies showed that the clamp drain technique up to 12 hours was safe and effective for blood loss reduction [9–13]. Therefore, in this prospective experimental study, we aimed to evaluate the effect of IA-TXA application with prolonged clamp drain technique on pharmacokinetics and systemic hemostasis.

Our data showed the systemic absorption after IA-TXA was independently affected with prolonged clamping drain method. Based on our findings, the systemic absorption of TXA was highest at 2 hours after application with drain clamp and gradually declined until 12 hours (Figure 1). Therefore, the drain-clamping time did not affect the systemic absorption in this study. This could be explained by blockage of TXA absorption due to an increase of clot formation on surgical bed and by consumption of the small concentration of IA-TXA (500 mg of TXA) into the intra-articular blood clot. The peak of serum concentration that was found in 2 hours after application of IA-TXA should be due to the absorption of TXA before the intra-articular clot formation and further metabolized in systemic circulation. After the clot formation was maximally established, an only minimal or no systemic absorption then occurred and, consequently, none of serum TXA level spikes was observed even with longer drain clamping applied. Moreover, this study also demonstrated that the TXA level in drainage fluid at the time of clamp release was not significantly different among groups (Table 2). This may imply that the synovial tissue did not play a role in the metabolism of IA-TXA for at least 12 hours, and low-dosage IA-TXA application was sufficient for inducing clot formation within joint space and stabilizing clot for at least 12 hours comparable with intravenous TXA administration [20].

Concerning the postoperative blood loss, there was no significant difference in any parameters on postoperative blood loss (Table 3). However, even without statistical significance, the lowest total hemoglobin loss and calculated total blood loss were found in 8 hours clamping drain group. Therefore, we concluded that, with our protocol, the small dose of IA-TXA for 500 mg of TXA with prolonged drain clamp up to 8 hours should be safe protocol in order to minimize blood loss without significant increase in systemic absorption.

Venous thrombosis becomes a main concern immediately after major orthopedic surgery. During TKR with tourniquet, venous stasis and endothelial injuries may also cause venous thrombosis [21]. The use of TXA may increase this risk. Several studies have measured biological markers of fibrinolysis (such as D-Dimers, plasmin, antiplasmin, and t-PA) and coagulation (e.g., prothrombin fragment 1.2, PAI-1) perioperatively in major orthopedic surgery. A reduced fibrinolytic activity may be associated with increased thrombotic risk [22, 23]. In this study, we used coagulation and fibrinolytic markers (as D-dimer, fibrinogen, plasmin inhibitor (PI), plasminogen (PLG), plasminogen activator inhibitor type 1 (PAI-1), and tissue plasminogen activator (t-PA)) to evaluate the systemic event after application of IA-TXA. Our results showed significant differences between groups in D-dimer (14 d, *p* = 0.01) and fibrinogen (time of clamp release, *p* = 0.02) (Table 4). However, none of radiographic evidences as detected by duplex ultrasonography on postoperative day 4 and clinical evidences of venous thrombosis during 1-year follow-up period was observed in this study. Therefore, this phenomenon should be caused by majority of intra-articular clot formation and clot lysis process.

PAI-1 and t-PA had also found a significant difference between groups, PAI-1 (time of clamp release, 24 hours, and 3 days, *p* = 0.002, 0.03, and 0.03, resp.) and t-PA (2 hours and time of clamp release, *p* = 0.05 and 0.02) (Table 4). This might be explained by a significant preoperative higher value due to patients' baseline status [24]. In fact, fibrinolysis with t-PA synthesis was located at the surgical site (lower limb), while venous samples were taken from the upper limbs. Theoretically, in the operated limb, fibrinolysis is activated by a local increase in t-PA released by the endothelium, leading in turn to clot destruction. As t-PA not bound to fibrin is rapidly inhibited by PAI, an increase in active t-PA should not be observed in the nonoperated limb.

This present study had some limitations. This study was carried out on a relatively small population; although our result showed no significant difference in systemic absorption related to prolonged clamp drain period, it still needed more study population to demonstrate the effect of prolonged clamping drain to any rare possible postoperative complications such as DVT or PE.

In conclusion, IA-TXA application in TKR with a small dose of 500 mg together with prolonged drain clamp up to 8 hours should be a safe protocol in order to minimize blood loss without significant increase in systemic absorption. The peak of serum concentration that was found in 2 hours after application of IA-TXA should be due to the absorption of TXA before the intra-articular clot formation and further metabolized in systemic circulation and no effect in intravascular clot formation.

## Figures and Tables

**Table 1:** Patients' characteristics data.

	Group						*p* value
	2 h	4 h	6 h	8 h	10 h	12 h	
Female gender^■^	4 (80)	2 (40)	4 (80)	4 (80)	5 (100)	3 (60)	0.36
Age, year^◆^	69 ± 7	72 ± 6	68 ± 7	67 ± 9	68 ± 4	74 ± 9	0.58
Height, cm^◆^	152 ± 5	155 ± 7	162 ± 11	153 ± 10	150 ± 5	158 ± 4	0.38
Weight, kg^◆^	61 ± 6	62 ± 6	67 ± 5	66 ± 10	67 ± 7	63 ± 4	0.68
BMI, kg/m^2◆^	27 ± 2	26 ± 2	27 ± 3	29 ± 3	30 ± 3	25 ± 1	0.16
ASA physical status, grade I/II	2/3	3/2	3/2	3/2	1/4	1/4	0.56
Right side^■^	2 (40)	3 (60)	3 (60)	5 (100)	4 (80)	2 (40)	0.32
Preoperative laboratory values^◆^							
Hemoglobin, g/dL	13.5 ± 0.8	13.5 ± 1.5	12.7 ± 1.3	12.3 ± 1.4	12.1 ± 0.7	14.0 ± 2.0	0.26
Platelet count, ×10^3^/mm^3^	267 ± 48	275 ± 70	267 ± 84	251 ± 40	283 ± 72	255 ± 83	0.97
Prothrombin time, sec	11.7 ± 0.5	11.6 ± 0.8	11.5 ± 0.2	11.7 ± 0.7	11.9 ± 1.3	11.6 ± 0.4	0.96
Partial thromboplastin time, sec	28.1 ± 2.4	32.4 ± 0.8	27.7 ± 4.6	27.7 ± 4.6	24.4 ± 1.8	28.3 ± 8.9	0.53
Thrombin time, sec	10.7 ± 0.3	10.8 ± 0.5	10.5 ± 0.5	10.8 ± 1.1	10.3 ± 0.5	10.4 ± 0.5	0.60
INR	1.02 ± 0.04	1.01 ± 0.07	1.00 ± 0.02	1.01 ± 0.06	1.04 ± 0.10	1.01 ± 0.03	0.95
D-dimer, ng/mL	209 ± 64	348 ± 253	190 ± 70	184 ± 84	289 ± 176	335 ± 183	0.37
Fibrinogen, mg/dL	310 ± 37	310 ± 79	357 ± 68	338 ± 108	434 ± 122	442 ± 138	0.21

■: value presented as number of subjects (percentage), ◆: value presented as mean ± standard deviation.

**Table 2:** Serum tranexamic acid (TXA) level and TXA level in drainage fluid.

	Group						*p* value
	2 h	4 h	6 h	8 h	10 h	12 h	
Serum TXA level, *μ*g/mL^◆^							
2 hours	12.8 ± 1.6	12.2 ± 2.6	15.0 ± 2.4	11.3 ± 2.1	13.2 ± 1.9	12.1 ± 2.3	0.16
Clamp release	12.8 ± 1.6	9.4 ± 3.3	6.4 ± 2.4	2.3 ± 0.5	2.0 ± 0.7	1.7 ± 1.0	<0.0001^**∗**^
24 hours	0.3 ± 0.1	0.5 ± 0.6	0.4 ± 0.2	0.2 ± 0.1	0.3 ± 0.1	0.3 ± 0.2	0.51
Drainage TXA level, *μ*g/mL^◆^	461.9 ± 181.3	856 ± 505.8	282.8 ± 142.9	530.9 ± 839.4	560.5 ± 466.7	736.5 ± 621.6	0.59

◆: value presented as mean ± standard deviation.

*∗*: significant difference among groups with *p* < 0.05.

**Table 3:** Blood loss outcome.

	Group						*p* value
	2 h	4 h	6 h	8 h	10 h	12 h	
Drainage blood loss, mL^◆^	418 ± 216	210 ± 117	266 ± 111	278 ± 118	238 ± 140	276 ± 163	0.35
Total hemoglobin loss, g/dL^◆^	2.5 ± 0.7	2.4 ± 0.8	2.0 ± 0.8	2.0 ± 0.5	2.2 ± 0.9	3.0 ± 0.8	0.37
Calculated total blood loss, mL^◆^	236 ± 105	245 ± 61	196 ± 106	154 ± 55	208 ± 112	277 ± 95	0.39

◆: value presented as mean ± standard deviation.

**Table 4:** *p* value of coagulation and fibrinolytic markers between each groups.

	*p* value between groups					
	Preop.	2 hours	C.R.	24 hours	3 days	14 days
Platelet count	0.97	0.77	0.69	0.52	0.68	0.92
Prothrombin time	0.96	0.92	0.83	0.86	0.12	0.62
Partial thromboplastin time	0.53	0.62	0.66	0.89	0.13	0.90
Thrombin time	0.60	0.99	0.75	0.64	0.47	0.91
INR	0.95	0.95	0.30	0.95	0.12	0.66
D-dimer	0.37	0.21	0.64	0.53	0.62	0.01^**∗**^
Fibrinogen level	0.21	0.28	0.02^**∗**^	0.13	0.38	1.00
Plasmin inhibitor	0.78	0.42	0.71	0.38	0.71	NA
Plasminogen	0.75	0.11	0.41	0.27	0.61	NA
Plasminogen activator inhibitor	0.02^**∗**^	0.20	0.004^**∗**^	0.05^**∗**^	0.06	NA
Tissue plasminogen activator	0.23	0.05^**∗**^	0.02^**∗**^	0.08	0.09	NA

Preop.: preoperative, C.R.; clamp release, and INR; international normalized ratio.

*∗*; Significant difference among groups with *p* < 0.05, NA; not available.
